# Effect of empagliflozin on ectopic fat stores and myocardial energetics in type 2 diabetes: the EMPACEF study

**DOI:** 10.1186/s12933-021-01237-2

**Published:** 2021-03-01

**Authors:** B. Gaborit, P. Ancel, A. E. Abdullah, F. Maurice, I. Abdesselam, A. Calen, A. Soghomonian, M. Houssays, I. Varlet, M. Eisinger, A. Lasbleiz, F. Peiretti, C. E. Bornet, Y. Lefur, L. Pini, S. Rapacchi, M. Bernard, N. Resseguier, P. Darmon, F. Kober, A. Dutour

**Affiliations:** 1 Aix Marseille Univ, INSERM, INRAE, C2VN Marseille, France; 2 Department of Endocrinology, Metabolic Diseases and Nutrition, Pôle ENDO, APHM, Hôpital Nord, Chemin Des Bourrely, 13915 Marseille cedex 20, France; 3 Aix Marseille Univ, CNRS, CRMBM, Marseille, France; 4 Assistance-Publique Hôpitaux de Marseille, Medical Evaluation Department, CIC-CPCET, 13005 Marseille, France; 5 Support Unit for Clinical Research and Economic Evaluation, Assistance Publique-Hôpitaux de Marseille, 13385 Marseille, France; 6 Aix-Marseille Univ, EA 3279 CEReSS–Health Service Research and Quality of Life Center, Marseille, France

**Keywords:** SGLT2 inhibitors, Epicardial adipose tissue, Ectopic fat, Myocardial energetics, Pcr/atp, ^31^P-MRS, MRI

## Abstract

**Background:**

Empagliflozin is a sodium-glucose cotransporter 2 (SGLT2) inhibitor that has demonstrated cardiovascular and renal protection in patients with type 2 diabetes (T2D). We hypothesized that empaglifozin (EMPA) could modulate ectopic fat stores and myocardial energetics in high-fat-high-sucrose (HFHS) diet mice and in type 2 diabetics (T2D).

**Methods:**

C57BL/6 HFHS mice (*n* = 24) and T2D subjects (*n* = 56) were randomly assigned to 12 weeks of treatment with EMPA (30 mg/kg in mice, 10 mg/day in humans) or with placebo. A 4.7 T or 3 T MRI with ^1^H-MRS evaluation–myocardial fat (primary endpoint) and liver fat content (LFC)–were performed at baseline and at 12 weeks. In humans, standard cardiac MRI was coupled with myocardial energetics (PCr/ATP) measured with ^31^P-MRS. Subcutaneous (SAT) abdominal, visceral (VAT), epicardial and pancreatic fat were also evaluated.

The primary efficacy endpoint was the change in epicardial fat volume between EMPA and placebo from baseline to 12 weeks. Secondary endpoints were the differences in PCr/ATP ratio, myocardial, liver and pancreatic fat content, SAT and VAT between groups at 12 weeks.

**Results:**

In mice fed HFHS, EMPA significantly improved glucose tolerance and increased blood ketone bodies (KB) and β-hydroxybutyrate levels (*p* < 0.05) compared to placebo. Mice fed HFHS had increased myocardial and liver fat content compared to standard diet mice. EMPA significantly attenuated liver fat content by 55%, (*p* < 0.001) but had no effect on myocardial fat.

In the human study, all the 56 patients had normal LV function with mean LVEF = 63.4 ± 7.9%. Compared to placebo, T2D patients treated with EMPA significantly lost weight (− 2.6 kg [− 1.2; − 3.7]) and improved their HbA1c by 0.88 ± 0.74%. Hematocrit and EPO levels were significantly increased in the EMPA group compared to placebo (*p* < 0.0001, *p* = 0.041). EMPA significantly increased glycosuria and plasma KB levels compared to placebo (*p* < 0.0001, *p* = 0.012, respectively), and significantly reduced liver fat content (− 27 ± 23 vs. − 2 ± 24%, *p* = 0.0005) and visceral fat (− 7.8% [− 15.3; − 5.6] vs. − 0.1% [− 1.1;6.5], *p* = 0.043), but had no effect on myocardial or epicardial fat. At 12 weeks, no significant change was observed in the myocardial PCr/ATP (*p* = 0.57 between groups).

**Conclusions:**

EMPA effectively reduced liver fat in mice and humans without changing epicardial, myocardial fat or myocardial energetics, rebutting the thrifty substrate hypothesis for cardiovascular protection of SGLT2 inhibitors.

*Trial registration* NCT, NCT03118336. Registered 18 April 2017, https://clinicaltrials.gov/ct2/show/NCT03118336

## Background

Sodium-glucose cotransporter 2 (SGLT2) inhibitors are a unique class of oral antidiabetic medications that reduce glucose reabsorption in the renal proximal tubules, thereby enhancing urinary glucose excretion. Large randomized controlled trials on the SGLT2 inhibitors, empagliflozin (EMPA-REG OUTCOME), canagliflozin (CANVAS PROGRAM), and dapagliflozin (DECLARE-TIMI 58) have been found to markedly reduce cardiovascular events and heart failure hospitalizations and to significantly improve renal outcomes in patients with type 2 diabetes (T2D) with a high or very high cardiovascular risk [1–3]. Multiple hypotheses have been proposed to explain the beneficial effects of SGLT2 inhibitors, which could be multifactorial, the most common one being the effect on diuresis/natriuresis [4]. In a hypertensive heart failure rat model, empagliflozin improved hemodynamics, attenuated intra-cardiac fibrosis and modulated genes related to fatty acid metabolism (PPAR and ACADM) [5]. In LPS-treated cardiomyocytes and macrophages and in LPS-treated mice, empagliflozin reduced inflammation, promoted the expression of anti-inflammatory M2 marker proteins, and activated AMPK, preventing ATP/ADP depletion [6]. In an experimental metabolic syndrome with heart failure with preserved ejection fraction (HFpEF) in the ZFS1 rat, empagliflozin improved endothelial function, cardiac remodeling, and senescence, and reduced perirenal fat [7]. However, the mechanisms involved in these early impressive cardiac benefits are still not completely understood. Type 2 diabetes is associated with an increase in ectopic fat deposition in the heart, liver and pancreas. One anatomic supplier of substrates to the myocardium is cardiac ectopic fat. No fascia separates epicardial adipose tissue adipocytes from cardiomyocytes and the coronary blood stream. Myocardial triglyceride content has been suggested to be an energy reserve for the myocardium in contexts of high energy demand [8]. Previous studies did not observe any significant association between the phosphocreatine (PCr)/adenosine triphosphate ratio, a recognized in vivo marker of myocardial energy metabolism and cardiac fat compartments [9]. We previously demonstrated that patients with T2D have increased epicardial and myocardial fat and that these ectopic fat stores could negatively affect left ventricular function and contribute to coronary artery disease (CAD) or heart failure [10, 11]. Here we examined whether SGLT2 inhibitors had an impact on ectopic fat stores and myocardial energetics in humans and mice. Impairment in myocardial high-energy phosphate metabolism and bioenergetics, characterized by a reduced cardiac phosphocreatine-to-ATP ratio (PCr/ATP), has been postulated to play a major role in the development and progression of heart failure [12, 13]. Adenosine triphosphate (ATP) is essential for normal cardiac function, including myofibrillar contraction, ion transport, and myocyte viability. Also, the PCr/ATP ratio, which is a powerful index of the energetic state of the heart [14] correlates with heart failure severity and is a strong predictor of cardiovascular mortality [15]. In the diabetic heart developing heart failure, metabolic flexibility of the heart allows oxidation of other substrates such as ketone bodies (KB), lactate and branched-chain amino acids. Interestingly, KB are more energetically efficient than other substrates, requiring the lowest oxygen consumption [16, 17]. Empagliflozin increases plasma ketone body levels, and it has therefore been hypothesized that a shift in energy substrate metabolism towards KB or an increased availability of ketones explains the positive cardiovascular outcomes in the EMPA-REG OUTCOME study. A recent study using ^11^C-palmitate and ^18^F-FDG PET/CT reported no change in myocardial FFA uptake but a significant 57% reduction in myocardial glucose uptake in T2D individuals treated with empagliflozin [18].

Multiple imaging modalities such as ^31^P-MRS and ^1^H-MRS allow cardiac energetics and ectopic fat stores to be measured in vivo noninvasively [19]. We investigated the effect of 12 weeks of empagliflozin treatment on ectopic fat stores (except for epicardial fat) in mice fed a high-fat high-sucrose diet (HFHS) and on ectopic fat stores and myocardial energetics (PCr/ATP) in T2D patients.

In the human study, the primary efficacy endpoint was the change in epicardial fat volume between EMPA and placebo from baseline to 12 weeks. Secondary end points were the differences in PCr/ATP ratio, myocardial, liver and pancreatic fat content, SAT and VAT between groups at 12 weeks.

## Methods

### Mouse study

#### Animals and experimental procedure

All the animal procedures were approved by the ethics committee of Aix-Marseille University and complied with the European Convention for the protection of animals used for experimental purposes. Animal MRI experiments were carried out on a 4.7T Bruker Biospec Avance system (Bruker, Ettlingen, Germany) dedicated to rodent exploration and equipped with a 70 mm birdcage TX resonator and a decoupled surface receive coil.

**Fig. 1:**
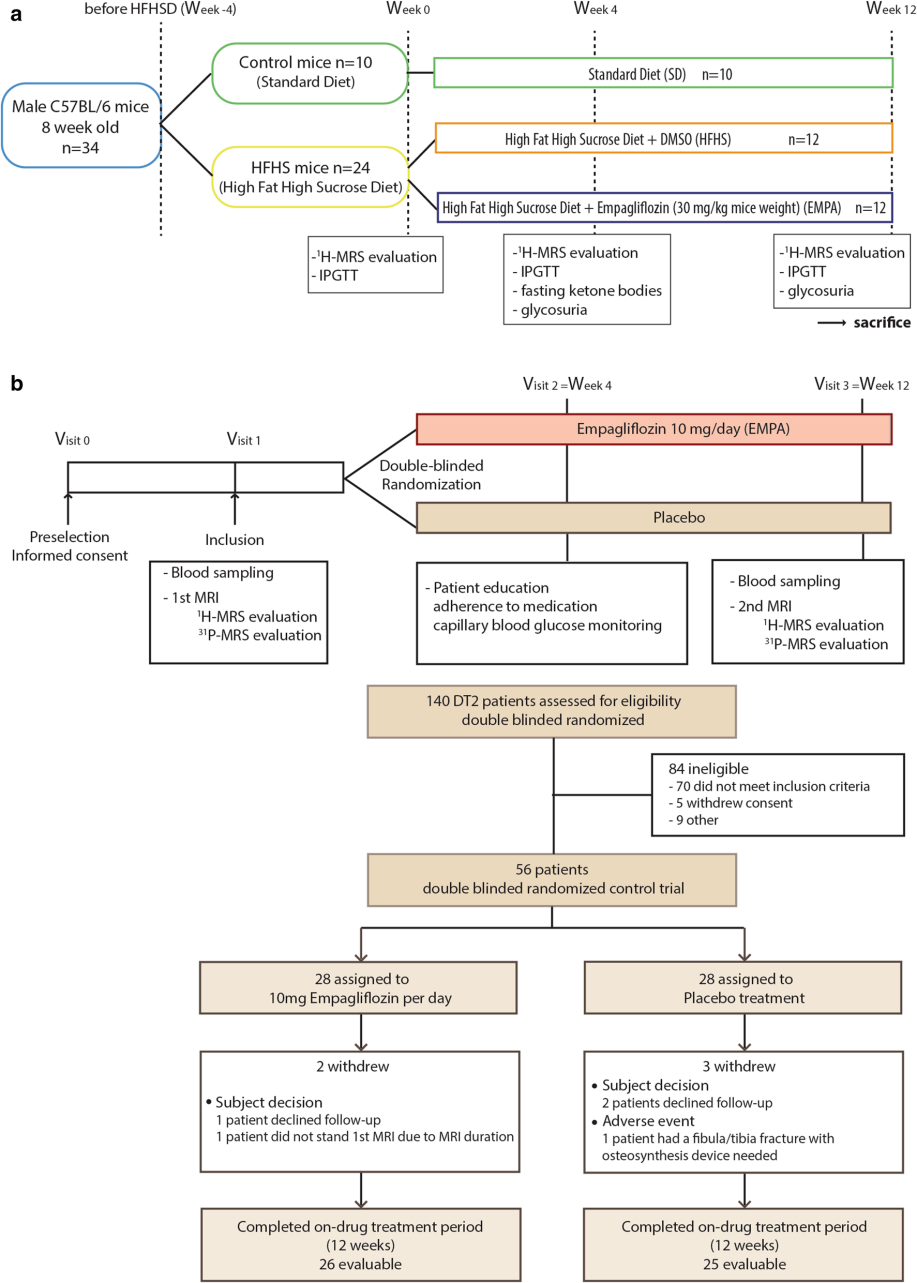
Study design and flow chart.** a** Mice design.** b** Human design. IPGTT, intraperitoneal glucose tolerance test; MRI, magnetic resonance imaging; MRS, magnetic resonance spectroscopy

Thirty-four C57BL/6 8-week-old male mice (Janvier, France) were housed in a controlled environment under standard laboratory conditions (12–12 h light–dark cycle, 22 °C). The mice were randomly divided into: Control (*n* = 10) with standard diet (#U8224G10R, Safe Diets, France) and HFHS (*n* = 24) diet (#U8978P Version 0019, Safe Diets, France) as previously described [20] (Fig. 1). At 4 weeks, metabolic status (intraperitoneal glucose tolerance test (IPGTT), glucagon, insulin and blood ketones) and MRI evaluation of ectopic fat stores (liver and myocardial, as rodents have no epicardial fat) were performed. The HFHS group was then randomly divided into two subgroups, EMPA (*n* = 12), with empagliflozin in water (30 mg/kg bodyweight + 0.1% DMSO (Sigma, USA)), and a placebo group HFHS (*n* = 12) with 0.1% DMSO in water. Metabolic status and MRI evaluation were performed after 4 and 12 weeks of treatment. At 24 weeks, mice were sacrificed, and tissues (heart, liver) collected (Additional file 1: Appendix S1).

### Human study

#### Study design

This randomized, parallel-group, double-blind, phase 3 trial was performed at the Endocrinology, Metabolic Diseases and Nutrition Department (Marseille, France) with a 1:1 allocation to treatment arms. The local ethics committee (CPP Ile-de-France VII/ANSM) approved all the trial procedures in compliance with the Declaration of Helsinki. The EMPACEF clinical trial was registered at clinicaltrials.gov (NCT03118336).

##### Patients

Subjects diagnosed with T2D, on stable antidiabetic medication, were recruited and randomly assigned to empagliflozin (10 mg/day) or placebo treatment. Before inclusion, all the patients gave their written informed consent.

Principal inclusion criteria were T2D (as defined by the World Health Organization criteria [21]), age ≥ 18 years, HbA1c 7–10%, renal function (MDRD) > 60 mL/min, on glucose-lowering therapy (metformin, sulfonylureas, glinides, dipeptidyl peptidase-4 (DPP-4) inhibitors, GLP-1 receptor agonists or basal insulin) at a stable dose for at least 3 months.

Participants were excluded if they had uncontrolled hyperglycemia at screening (fasting blood glucose FBG ≥ 240 mg/dL, were pregnant/breastfeeding or treated with ongoing corticoids or thiazolidinedione, had acute coronary syndrome or unstable angina during the last 3 months, or stage 4 lower limb peripheral artery disease (PAD) or previous history of lower limb amputations, any contraindication to magnetic resonance imaging, or more than 5% total body weight loss within the last month.

##### Randomization and masking

Participants were randomly assigned by a stratified computed randomization procedure accounting for sex, age, and BMI to the empagliflozin group (EMPA) or placebo and were masked to the treatment assignment. Randomization and assignment to the double-blind trial was done by the central pharmacist. Blinding of investigators and patients was ensured by supplying EMPA and placebo tablets with identical appearance and packaging. Unblinding was performed by blinded research assistants after all the MRI post-processing measurements had been made.

##### Procedures

Patients received one individual dietary counseling session before the baseline visit, according to American Diabetes Association recommendations [22]. Patient education, clinical examination with anthropometrics (height, weight, BMI, waist circumference), systolic and diastolic blood pressure blood sampling (FBG, lipid profile, uricemia, hematocrit, erythropoietin, ketonemia, glycosuria, PAI-1, ferritin, liver enzymes) treatment safety, and adherence were assessed at weeks 0 (Visit 1 V1), 4 (V2) and 12 (V3).

Enrolled patients were allocated to one treatment arm, empagliflozin 10 mg once daily or matching placebo orally (Boehringer Ingelheim, Ingelheim/Rhein, Germany) for 12 weeks.

##### Magnetic resonance imaging

All human MR imaging and spectroscopy was carried out on a 70 cm diameter 3 T MR system (Verio, Siemens Healthineers, Erlangen, Germany) equipped with a 32-element phased array coil. Visceral abdominal adipose tissue area was measured with a 5 mm transverse slice at L4-L5 intervertebral disk level, using a 3-point Dixon sequence at baseline (week 0) and 12 weeks, as previously described [23].

Liver fat, pancreatic fat and myocardial triglyceride content were quantified with ^1^H-spectroscopy, using a single voxel PRESS sequence, as previously described [11, 24]. Epicardial fat volume and cardiac function were assessed, as previously described [10], using a multislice steady-state free precession cine sequence in short-axis view that covered the LV from base to apex and were manually contoured (epicardial and endocardial left ventricular contours) using dedicated post-processing software (Argus, Siemens Medical Solutions). Left ventricular ejection fraction (LVEF) and LV mass were calculated with Argus software. LV mass was indexed with body surface area, as previously described [10].

##### ^31^P nuclear magnetic resonance spectroscopy

A ^31^P Surface Heart Coil (Rapid Biomedical GmbH, Rimpar, Germany, O-XQ-030–01,605 V01) with a working frequency of 49.9 Hz was used for radiofrequency transmit and receive. A 3D chemical shift imaging (CSI) sequence was used to acquire phosphorus MR spectroscopic imaging data (TR 1300 ms, flip angle 70 degrees, Hamming acquisition *k*-space weighting with six excitations in the center of *k*-space). The sequence was run without ECG or respiratory gating. The radiofrequency voltage necessary to produce a flip angle of 90 degrees in the centrally placed PPA tube (10 mL of 0.6 M phenyl phosphoric acid) on the surface coil was determined beforehand using a calibration scan. Spectroscopic acquisition time was 34 min owing to a relatively small voxel size (15 × 15 × 24 mm).

^31^P spectra were analyzed according to Bottomley [25] using software developed in-house [26] running in the IDL framework (Interactive Data Language; ITT Visual Solutions, Boulder, CO, USA). Voxels were placed in the mid inter-ventricular septal wall across a stack of short-axis images, starting from the base of the heart in a slice where left and right ventricles were visually comparable in terms of surface area (approximately at LV mid-level).

A ^31^P SMR spectrum was obtained from each voxel after spectral filtering at 15 Hz and adjusting both phase and frequency shift to obtain a PCr peak at 0 ppm. Spectra were later fitted using AMARES (Advanced Method for Accurate, Robust, Efficient Spectral Fitting) [27]. The ratio was corrected for the contribution of ATP in the left ventricular blood to the myocardial ATP resonance [25]. A previously built B1^+^ map of the surface coil was used to estimate the local flip angle at the location of each voxel. Signal amplitudes were then corrected for partial saturation using this estimated local flip angle and literature T1 values of the corresponding resonances [28]. The ratio of the corrected PCr to γ-ATP signal amplitudes was used to generate a PCr/ATP concentration ratio [29, 30].

##### Human study outcomes

The primary efficacy endpoint was defined as the difference in change in epicardial fat volume between EMPA and placebo from baseline to 12 weeks of treatment. Secondary endpoints were the differences in changes in PCr/ATP ratio, myocardial, liver and pancreatic fat content, subcutaneous and visceral abdominal fat between EMPA and placebo from baseline to 12 weeks.

##### Power calculation

The sample size calculation was based on our previous work on exenatide treatment and ectopic fat stores [24]. Sample size calculation was performed under the following hypotheses: (i) a difference between EMPA group and placebo group regarding the primary endpoint of 7.2, (ii) a standard deviation for both groups regarding the primary endpoint of 7.7, (iii) an allocation ratio of 1:1, (iv) a power of 0.8 and a bilateral alpha risk of 0.05. A total of 38 patients were required (19 in each group). Allowing for a risk of loss to follow-up of 10% and technical MRI issues, the sample size was increased to 56.

### Statistical analyses

Statistical analyses were performed using Prism v8 (Graphpad, San Diego, CA, USA) or SPSS v23 (Chicago, USA) and the significance threshold was *p* = 0.05. Results are presented as means ± standard deviation or median (25th, 75th percentile). Normality of variables was analyzed by the Shapiro–Wilk normality test. Datasets were compared using paired Student’s *t*-tests for normal distribution, with either a Mann–Whitney *U*-test or Wilcoxon signed rank test if not parametric. Two-way repeated measures ANOVA was performed with treatment as factor to test time and group interaction. Intention-to-treat analysis was performed for primary outcome assessment using the Last Observation Carried Forward (LOCF) method. Per-protocol analysis was then performed.

## Results

### Mouse study

**Fig. 2:**
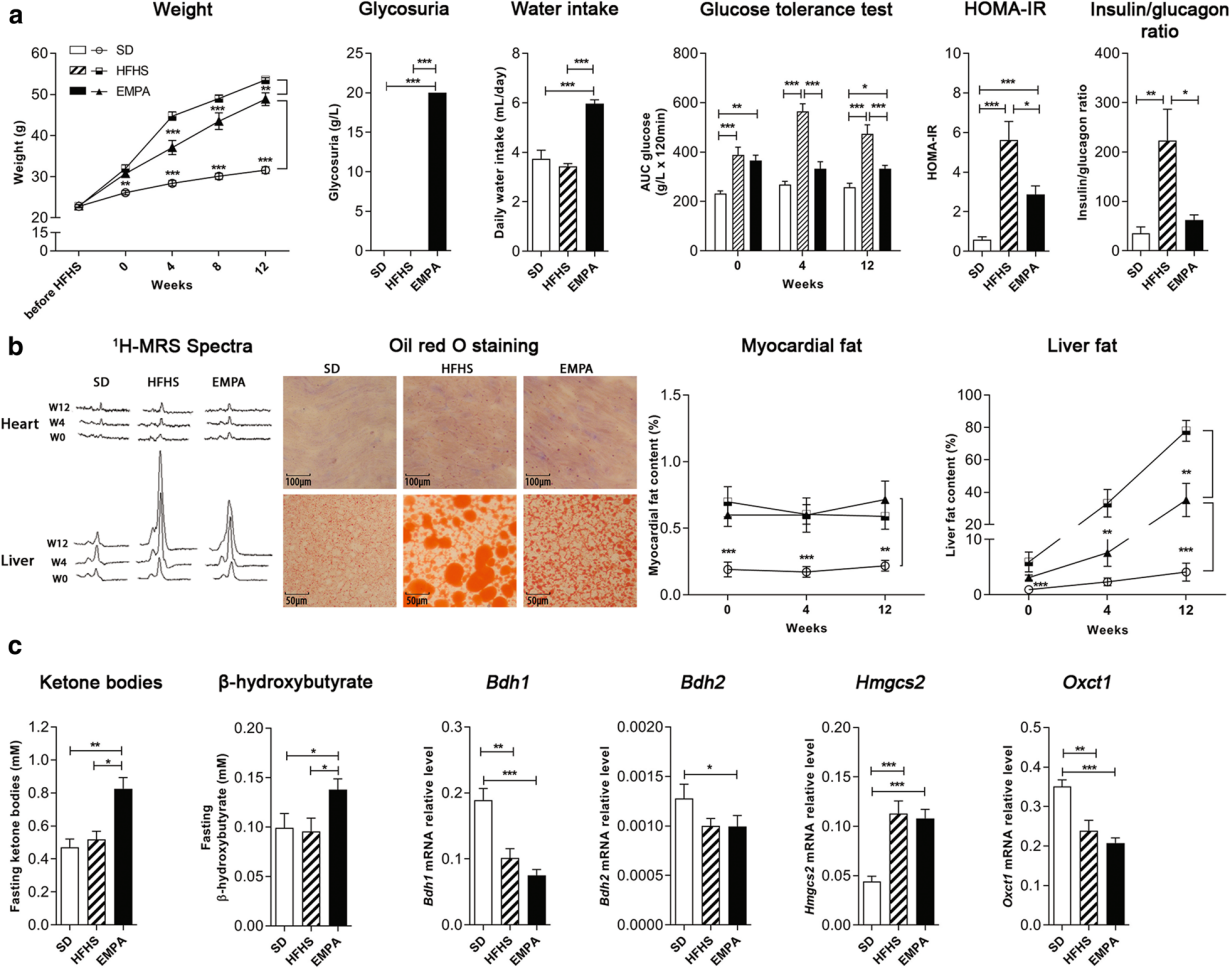
Changes in metabolic profile and ectopic fat stores at 12 weeks of treatment in mice.** a** Metabolic profile: Weight of mice during 12 weeks of standard diet (SD), High Fat High Sucrose diet (HFHS) or HFHS + empagliflozin (EMPA); glycosuria at 4 and 12 weeks in SD, HFHS and EMPA mice; mean daily water intake (mL/day) in SD, HFHS and EMPA mice measured every 2 days for 12 weeks; intraperitoneal glucose tolerance test (IPGTT) in SD, HFHS and EMPA mice measured at week 0, 4 and 12 of empagliflozin treatment; HOMA-IR index and insulin/glucagon ratio of SD, HFHS and EMPA mice measured at 12 weeks.** b** Ectopic fat stores: Evolution of typical ^1^H-MRS spectra (triglycerides peak at 1.3 ppm) acquired in the heart or liver of SD, HFHS and EMPA mice at week 0, 4 and 12; Representative Oil-Red-O stain images (*n* = 10 images per mouse) of lipid droplets in heart and liver from SD, HFHS or EMPA mice; scale bars, 100 μm for heart and 50 µm for liver.** c** Ketone pathways: Ketone bodies and fasting β-hydroxybutyrate of SD, HFHS and EMPA mice measured at 4 weeks; RT-qPCR analyses of genes coding for enzymes involved in β-oxidation pathway (*Bdh1, Bdh2, Hmgcs2, Oxct1*) of SD, HFHS and EMPA mice after 12 weeks. *SD (n* = 10), HFHS (*n* = 12), EMPA (*n* = 12). Data represent mean values ± SEM. Differences between SD, HFHS and EMPA were determined by nonparametric unpaired Mann–Whitney comparative tests or ANOVA with Tukey's post hoc test; *p < 0.05, **p < 0.01, ***p < 0.001 (from post hoc test)

Mice fed HFHS became obese and glucose-intolerant at 4 weeks compared to wild-type mice fed standard diet (Fig. 2). As expected, empagliflozin treatment significantly reduced weight gain at 4 weeks (*p* < 0.001), increased daily water intake (*p* < 0.001), blood ketone bodies and β-hydroxybutyrate levels (*p* < 0.05) and glycosuria (*p* < 0.001) compared to HFHS mice fed placebo. EMPA treatment significantly improved glucose tolerance in the iPGTT test at 4 and 12 weeks, and decreased insulin/glucagon basal ratio at 12 weeks compared to HFHS mice (*p* < 0.001) (Fig. 2).

Mice fed HFHS had increased myocardial and liver fat content measured in vivo with ^1^H-MRS at 4 weeks compared to wild-type mice fed standard diet (Fig. 2). EMPA treatment significantly attenuated liver fat content (LFC) by 55%, compared to HFHS mice: LFC: 35.2 ± 10.3% EMPA vs. 77.9 ± 6.5 HFHS, *p* < 0.001) but had no effect on myocardial fat (*p* = NS). After sacrifice, oil-red-O staining showed a protective effect of EMPA treatment on liver neutral lipid content, but no effect on myocardial triglyceride accumulation compared to HFHS mice.

Of note, mice fed HFHS displayed a reduced oxidative capacity, as expressed by a significant decrease in the enzymes involved in the KB metabolism bdh1, bdh2, hmgcs2 and oxct1 mRNA levels, compared to wild-type mice (*p* < 0.05). Empagliflozin treatment did not modify the expression of these genes compared to HFHS mice (Fig. 2).

### Human study

Of the 140 T2D patients screened for the study, 84 were ineligible (Fig. 1) and 56 were included and randomly assigned to EMPA (*n* = 28) or placebo (*n* = 28), and received at least one dose of the study medication. Of all the randomized patients, only 51 completed the trial, (owing especially to MRI issues), and had complete ^1^H-MRS (Fig. 1). Owing to technical issues, only 34 patients (18 in the EMPA and 16 in the placebo group) had the two ^31^P-MRS evaluations. In the placebo group, there was one major adverse event declared with an accidental fibula/tibia fracture that needed osteosynthesis and contraindicated the patient for the second MRI.

### Patient characteristics

The mean age of the study population was 56.9 ± 9.6 years, with mean BMI 34.9 ± 6.0 kg/m^2^, HbA1c 8.13 ± 1.05% and mean disease duration 11.1 ± 6.7 years. Baseline anthropometric and clinical characteristics were all comparable between EMPA and placebo (Table 1). Physical activity did not differ between groups either at baseline or from baseline to 12 weeks.

**Table 1:** Baseline characteristics of the study population at baseline

	Placebo (*n* = 25)	Empa (*n* = 26)	*p*
Age (years)	58.6 ± 9.2	57.0 ± 10.1	0.483
Sex ratio (M, %)	10 (40)	10 (38)	> 0.999
BMI (kg/m^2^)	34.1 [30.5;39.5]	32.6 [31.8;36.6]	0.644
Diabetes duration (years)	9 [6;18]	9.5 [7;16.3]	0.862
HbA1c (%)	8.06 ± 1.10	8.20 ± 1.01	0.653
Hypertension (*n*, %)	13 (52)	19 (73)	0.153
Dyslipidemia (*n*, %)	19 (76)	18 (69)	0.755
Smoking status active (*n*, %)	5 (20)	5 (19)	> 0.999
History of CAD/PAD/stroke	7 (28)	9 (35)	0.765
Very high risk (*n*, %)^a^	19 (76%)	20 (77%)	> 0.999

BMI: body mass index; CAD: coronary artery disease; PAD: peripheral artery disease; ESC: European Society of Cardiology

^a^Cardiovascular risk according to *European Society of Cardiology* guidelines[60]

### Intention-to-treat analysis

#### Effect of EMPA on body composition and metabolic parameters

EMPA resulted in a significant weight loss of − 2.6 kg [− 3.7; − 1.2] at V3 (p < 0.0001) (Table 2). EMPA significantly improved glycemic control with a significant decrease in fasting blood glucose (− 2.24 ± 2.30 mmol (*p* < 0.0001 for time) and HbA1c by 0.88 ± 0.74% (*p* < 0.0001 time). Compared to placebo, these improvements were significant (*p* = 0.006, and *p* = 0.003 respectively). A significant decrease in uric acid was observed in the EMPA group compared to the placebo group (*p* = 0.005).

**Table 2:** Baseline characteristics and changes in parameters after 12 weeks of treatment

	Placebo (*n* = 25)			Empa (*n* = 26)			Difference between groups
	Baseline	Posttreatment	*p*	Baseline	Posttreatment	*p*	*p*
Clinical parameters							
Systolic blood pressure (mm Hg)	124 ± 16	124 ± 13	0.9395	125 ± 9	124 ± 9	0.5717	0.7109
Diastolic blood pressure (mm Hg)	74 ± 11	73 ± 10	0.8425	71 ± 8	71 ± 10	0.9717	0.9063
Weight (kg)	93 [83;105]	94 [82;104]	0.8882	91 [81;105]	89 [80;102]	< *0.0001*	*0.0047*
BMI (kg/m^2^)	34.1 [30.4;39.5]	33.2 [30.3;38.3]	0.8851	32.6 [31.8;36.5]	32 [30.6;35.3]	< *0.0001*	*0.0045*
Waist circumference (cm)	113 [103;124]	112 [102;121]	0.2623	114 [103;120]	113 [101;117]	*0.0360*	0.4266
Biological parameters							
Fasting blood glucose (mmol/L)	9.22 ± 2.49	8.74 ± 2.42	0.2516	9.59 ± 2.44	7.35 ± 1.73	< *0.0001*	*0.0063*
HbA1c (%)	8.1 ± 1.1	7.8 ± 0.9	0.0605	8.1 ± 1.0	7.3 ± 0.6	< *0.0001*	*0.0033*
Total cholesterol (g/L)	1.72 ± 0.40	1.68 ± 0.35	0.5340	1.57 ± 0.37	1.57 ± 0.44	0.8655	0.5111
HDL-cholesterol (g/L)	0.41 [0.32;0.45]	0.42 [0.33;0.5]	0.7394	0.40 [0.36;0.50]	0.46 [0.38;0.53]	*0.0123*	0.1359
LDL-cholesterol (g/L)	0.92 ± 0.25	0.92 ± 0.29	0.9668	0.83 ± 0.30	0.84 ± 0.34	0.6199	0.7940
Triglycerides (g/L)	1.56 [1.11;2.13]	1.47 [1.01;2.35]	0.9881	1.48 [0.94;1.97]	1.27 [0.84;1.54]	*0.0451*	0.6447
Uricemia (µmol/L)	284.6 ± 81.1	296.0 ± 87.8	0.3391	301.8 ± 82.3	265.7 ± 67.1	*0.0052*	*0.0062*
Ferritinemia	65.0 [15.0;158.9]	60.2 [16.4;126.5]	0.8462	75.7 [34.3;133.0]	48.6 [19.9;92.3]	< *0.0001*	*0.0445*
hs-CRP (mg/L)	3.7 [1.8;5.1]	3.4 [1.7;6.1]	0.1058	2.3 [1.0;5.6]	2.3 [1;5.3]	0.1349	0.2044
PAI-1 (UI/L)	41 [11;79]	36.5 [10.2;72]	0.3938	24 [15.2;39.2]	16 [8.5;28.5]	0.1396	0.4318
AST (UI/L)	23 [20;32]	24 [18;33]	0.6062	23 [20;33]	23 [19;32]	0.2339	0.1219
ALT (UI/L)	33 [23;54]	34 [23;44]	0.3178	30 [25;42]	28 [18;44]	*0.0302*	0.0705
GGT (UI/L)	42 [30;76]	43 [26;63]	0.3698	36 [26;47]	28 [24;42]	*0.0002*	0.2219
Hematocrit	0.38 [0.35;0.41]	0.38 [0.36;0.43]	0.5520	0.40 [0.37;0.43]	0.43 [0.40;0.46]	< *0.0001*	*0.0001*
Reticulocytes (%)	1.4 [1.2;1.8]	1.5 [1.1;1.7]	0.4463	1.5 [1.2;1.8]	1.3 [1.2;1.6]	*0.0030*	*0.0027*
EPO (mU/mL)	13 [8.5;18]	11 [8.2;16.5]	0.1709	8.7 [4.9;14]	9.2 [5.9;13.7]	0.1789	*0.0412*
Glycosuria (mmol/L)	0.75 [0.31;3.13]	1.06 [0.36;5.72]	0.6434	1.13 [0.56;12.83]	193.3 [125.4;256.7]	< *0.0001*	< *0.0001*
MRI parameters							
Ejection fraction (%)	63.7 ± 8.3	63.3 ± 7.7	0.6598	63.1 ± 8.2	63.3 ± 7.2	0.8515	0.6515
LVM (g)	129 [107;149]	124 [107;151]	0.2217	117 [93;150]	110 [97;142]	0.5153	0.9707
LVMi (g)	64 [59;72]	64 [57;69]	0.1429	58 [51;66]	58 [52;64]	0.3802	0.9545
E/A ratio	1.26 [1.07;1.49]	1.17 [0.85;1.44]	0.3289	1.14 [0.90;1.71]	1.08 [0.92;1.62]	0.5619	0.3923

BMI body mass index; PAI-1 plasminogen activator inhibitor-1; EPO: erythropoietin; LVEF left ventricular ejection fraction; LVM left ventricular mass; LVMi left ventricular mass indexed to body surface area

#### Effect of EMPA on epicardial fat volume

All the patients had normal LV function with mean LVEF = 63.5 ± 8.2%. We observed no significant change in epicardial fat volume from baseline to 12 weeks in the EMPA group (V1 108.5 ± 31.8 vs. V3 106.9 ± 31.8 mL *p* = 0.09) and in the placebo group (V1 105.8 ± 21.3 vs. 105.3 ± 20.7 mL at V3, *p* = 0.34) (*p* = 0.88 for the difference between groups). No change in LV mass, LVMi, or LVEF was found at 12 weeks between groups (Table 2).

#### Effect of EMPA on myocardial energetics

**Fig. 3:**
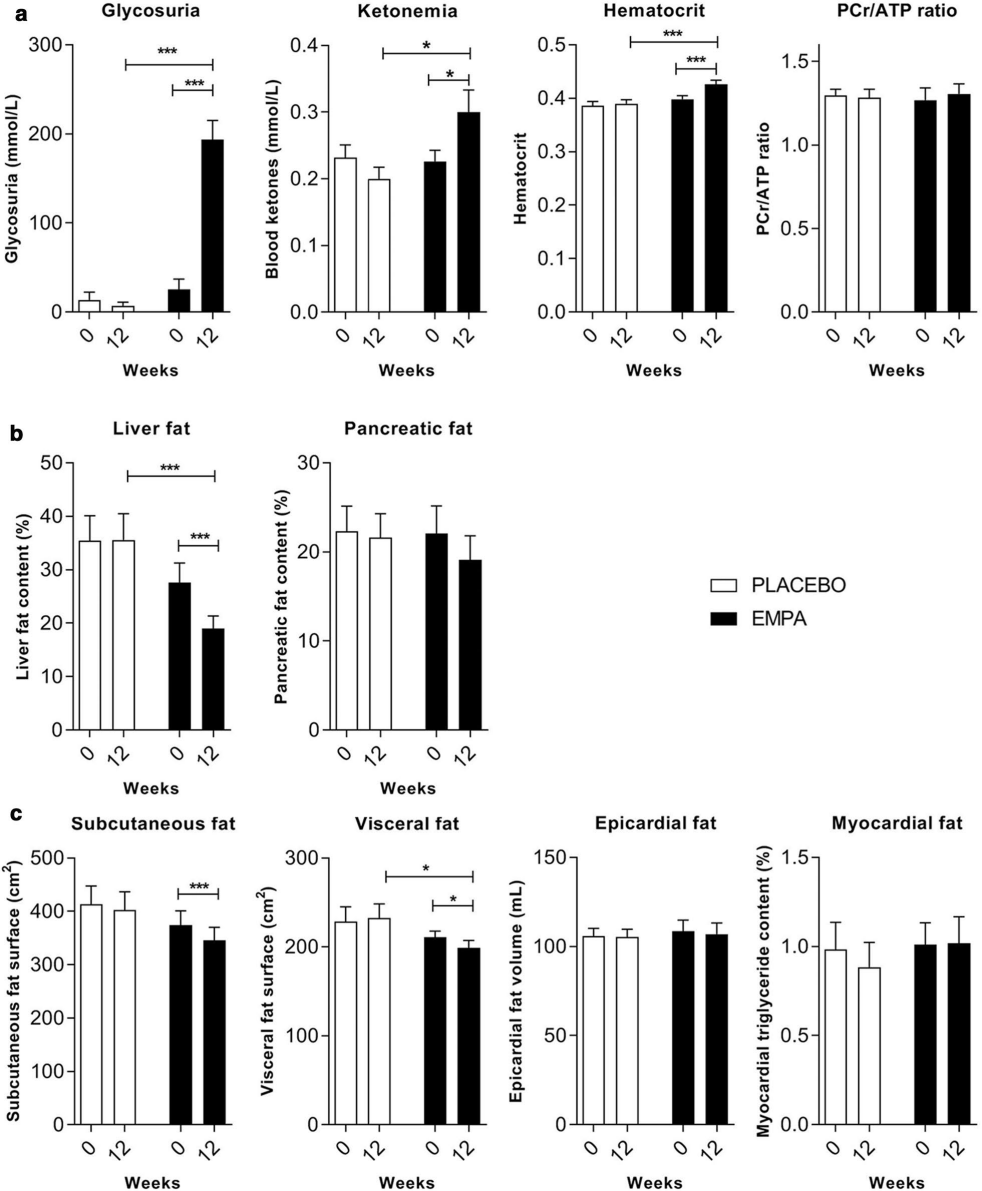
Changes in myocardial energetics, metabolic profile and ectopic fat stores at 12 weeks of treatment in humans. **a** Changes in glycosuria, blood ketone body levels, hematocrit and PCr/ATP ratio obtained by ^31^P-magnetic resonance spectroscopy in placebo and EMPA group. **b** Liver and pancreatic fat content assessed with ^1^H-magnetic resonance spectroscopy change between baseline and 12 weeks in placebo and EMPA group. **c** Visceral and subcutaneous abdominal fat change measured at L4-L5 intervertebral disk level; Epicardial fat volume assessed on cine MRI short axis slices and myocardial fat assessed with ^1^H-magnetic resonance spectroscopy at the interventricular septum with a single-voxel PRESS sequence. Placebo (*n* = 16), EMPA (*n* = 18). Data represent mean values ± SEM. Differences between baseline and 12 weeks were determined by paired *t*-test/Wilcoxon test and differences between groups with ANOVA; *p < 0.05, **p < 0.01, ***p < 0.001

As expected, EMPA treatment significantly increased glycosuria and KB levels (Fig. 3a) compared to placebo (*p* < 0.0001, *p* = 0.01, respectively). At 12 weeks, no significant change was observed in the PCr/ATP ratio either in the EMPA, or in the placebo group (Fig. 3a). Of note, hematocrit and EPO were significantly increased in the EMPA group compared to placebo (*p* = 0.0001 and *p* = 0.04, respectively) (Table 2).

#### Effect of EMPA on other ectopic fat stores

All the patients except for two in the placebo group had NAFLD (LFC > 5%) at baseline. LFC was comparable between groups (*p* = 0.25) at baseline and markedly decreased in the EMPA group by 27% from 22.5 [12.6–41.1] to 19.9 [8.49–28.3], compared to placebo by 2% from 31.5 [18.5–54.9] to 29.6 [16.7–55.7] *p* < 0.0001 for time, and *p* = 0.0005 for the difference between groups (Fig. 3b). This decrease was accompanied by a significant decrease in liver enzymes ALT and GGT in the EMPA group compared to placebo (for time *p* = 0.03, *p* = 0.0002 respectively, but this did not reach significance for the difference between groups, *p* = 0.07 and *p* = 0.22 respectively) (Table 1). Weight loss ≥ 5% occurred in 38% of patients on EMPA and in 12% of patients on placebo (*p* = 0.052). The LFC change was significantly correlated with weight loss (*r* = 0.39, *p* = 0.006). Patients in the EMPA group exhibited significant reduction of visceral abdominal fat (211.2 ± 31.4 at V1 to 199.1 ± 38.7 cm^2^ at V3, *p* = 0.04), compared to patients in the placebo group (228.7 ± 74.1 at V1 to 232.3 ± 71.6 cm^2^ at V3, *p* = 0.46), *p* = 0.04 between groups (Fig. 3c). Interestingly, no significant difference between groups was observed for subcutaneous fat, myocardial fat or pancreatic fat (Fig. 3b, c).

#### Per-protocol analysis

Per-protocol analysis confirmed the results obtained in ITT analysis with no significant reduction of EAT, myocardial, or pancreatic fat, but a significant reduction of visceral fat and LFC (EMPA *n* = 25 and placebo *n* = 23 per group *p* = 0.04 and *p* = 0.0011, respectively).

## Discussion

This trial provides evidence that empagliflozin effectively reduces liver steatosis in mice and humans compared with placebo but has no immediate effects on cardiac ectopic fat or myocardial energetics. Interestingly, these effects occurred as early as 12 weeks after treatment initiation in the presence of moderate weight loss and significant improvement in glycemic profile.

### Twelve weeks of empagliflozin treatment did not modulate epicardial fat volume in humans

Recently, there has been growing interest in how SGLT2 inhibitors reduce cardiovascular outcomes in patients with T2D [19]. However, few mechanisms of action have been robustly investigated. Novel multiple cardiac imaging modalities combining ^1^H-MRS and ^1^P-MRS enabled us to test the appealing hypothesis that empagliflozin could modulate ectopic fat stores such as cardiac ectopic fat (epicardial, myocardial fat), pancreatic fat or myocardial energetics (as assessed by PCr/ATP ratio). Epicardial fat volume mobilization was the primary endpoint of the human study.

Epicardial adipose tissue (EAT) is a visceral fat deposit located between the visceral pericardium and the myocardium. However, unlike visceral abdominal adipose tissue, it has predominantly local effects, making up less than 1% of total fat mass [8]. EAT has a unique anatomical proximity to the heart. As there is no dividing fascia plane between EAT, coronary arteries adventitia and myocardium, there can be cellular crosstalk and functional relationships between ectopic adipocytes, myocytes and cells of the vascular wall. Free fatty acids (FFAs), proinflammatory cytokines (IL-6, IL-1β, IL-8, sPLA2-IIA), vasoactive entities (angiotensin) adipofibrokines (Activin-A) and growth factors (TNF-α, VEGF) are likely to diffuse from EAT to the lumen of coronary arteries. The amount of EAT has been shown to predict fatal and non-fatal cardiac outcomes at 8 years in high-risk cardiovascular patients and has been associated with plaque vulnerability, myocardial infarction, unstable angina, coronary flow reserve, and stent restenosis [31, 32]. This study showed no effect of empagliflozin on epicardial fat volume in high-risk patients with T2D and normal LVEF. Epicardial fat was not measured in the mouse study because rodents have no EAT [8]. Few studies have evaluated the effect of SGLT2 inhibitors on epicardial fat volume [33–36]. These studies were observational, open-label, single-arm with multiple imaging modalities, and yielded conflicting results. To our knowledge, this is the first randomized, placebo-controlled clinical trial to examine the effect of empagliflozin on epicardial fat volume assessed with the more accurate non-invasive method (3 T MRI) in T2D subjects. In a lipodystrophic mouse model of diabetic cardiomyopathy (seipin-knockout mice with no lipotoxic hallmarks and no intramyocardial lipid accumulation), Joubert et al*.* showed a beneficial effect of dapagliflozin on cardiac dysfunction with improvement of myocardial glucose uptake, and decreased activation of the hexosamine pathway (O-GlcNacylated protein levels), suggesting that SGLT2 inhibitors could act on glucotoxicity rather than lipotoxicity mechanisms. Interestingly, whereas the heart does not express SGLT2, Diaz-Rodriguez et al*.* reported SGLT2 expression in epicardial adipose tissue, increased in vitro glucose uptake and GLUT4, reduced secretion of proinflammatory chemokines, and improved differentiation of EAT cells with dapagliflozin in EAT of patients undergoing open heart surgery (30% T2D, and 50% with CAD) [37]. The lack of change in epicardial fat volume observed in our study does not imply no change in molecular characteristics of EAT with SGLT2 inhibition that could influence heart function. This change in EAT phenotype might contribute at least to a decreased adverse remodeling of the overloaded diabetic heart.

### Twelve weeks of empagliflozin treatment did not change myocardial fat or left ventricular function

We observed no change in LV mass or E/A ratio at 12 weeks between groups, in contrast to the EMPA-HEART study, which reported a significant decrease in LV mass indexed to body surface area in patients with T2D and CAD and treated with empagliflozin after 6 months, suggesting that the improvement of LV function could be delayed and may not be sufficient to explain early cardiovascular benefits [38]. In db/db mice, Moellmann et al*.* showed that improvement of diastolic function could be mediated by reduced spontaneous diastolic sarcoplasmic reticulum calcium release, independently of changes in cardiac ketone metabolism [39]. Our results are consistent with the open-label trial of Hsu et al*.,* who found no effect of 6 months of empagliflozin treatment on LV function, pericardial myocardial fat, myocardial fibrosis or LV mass [34]. Likewise, this study reported no effect of empagliflozin on myocardial triglyceride content at 12 weeks.

### Twelve weeks of empagliflozin treatment did not change pancreatic fat content

SGLT-2 inhibitors are known to improve β cell function and insulin sensitivity in T2D [40]. Previous studies have suggested that increased pancreatic fat deposition (non-alcoholic fatty pancreas disease) could contribute to β cell dysfunction and could sustain insulin resistance [41]. We previously demonstrated that patients with T2D have an increased ectopic fat storage in the pancreas compared to obese nondiabetics and lean subjects [42]. To our knowledge, this study is innovative in that it is the first one reporting no effect of an SGLT2 inhibitor on pancreatic fat content in humans, as measured with ^1^H-MRS. With this technique, we could not differentiate intralobular or parenchymal fat in humans in vivo, as previously described by others with modified DIXON (mDIXON) water images and mDIXON MRI fat fraction method [43]. We did not assess pancreatic fat in mice because of challenging pancreas localization with ^1^H-MRS. This exploratory analysis now needs to be confirmed in larger long-term clinical trials dedicated to pancreas morphological features and endocrine pancreas function [44, 45].

### Twelve weeks of empagliflozin treatment greatly reduced liver fat content in mice and humans

This study showed a significant effect of empagliflozin on liver fat in mice and humans. In Otsuka Long-Evans Tokushima fatty rats, Kim et al. [46] had already shown significant reduction of hepatic steatosis with empagliflozin and a decrease in lipogenesis and gluconeogenesis gene expression, and increased SIRT1 and AMPK expression in the liver. In an Amylin liver non-alcoholic steato-hepatitis (NASH) model, Honda et al*.* [47] also demonstrated that ipragliflozin (40 mg/kg/d) for 8 weeks improved insulin resistance and liver injury, decreased hepatic lipid content, areas of fibrosis and also increased lipid outflow from the liver at 8 weeks of treatment. Other animal studies point to a reduction in hepatic inflammatory cytokines and oxidative stress or an increase in ketone body metabolism with SGLT-2 inhibitors [48]. However, this mouse study showed no effect of empagliflozin on genes coding for enzymes involved in KB metabolism. An effect of empagliflozin on liver fat content may be also explained by a negative energy balance via substantial urine glucose loss and with an increase in fatty acid oxidation that might be promoted by a decreased insulin/glucagon ratio [49]. This human study revealed that 96% of the studied population had liver steatosis (as defined by LFC > 5%) at baseline, underlining the extreme prevalence of NAFLD in T2D even in patients with normal transaminases levels (12,16,26). The effect of empagliflozin on liver fat content occurred in parallel to the decline in body weight during SGLT2 inhibitor treatment. We and others previously demonstrated that liver fat is the most rapidly mobilized ectopic fat store during nutritional, medical or surgery-induced weight loss interventions (27). Also, the weight reduction ≥ 5% observed in only 38% of patients on EMPA suggests that even minor weight loss might have an impact on liver fat content. These results are consistent with the E-LIFT open-label trial in which empagliflozin 10 mg significantly decreased MRI-derived liver proton density fat fraction (PDFF) compared to patients not treated with EMPA (28), and to the results of Kahl et al*.* [50] who reported a significant effect of empagliflozin 25 mg compared to placebo in T2D patients with excellent glycemic control and short disease duration. Other studies have yielded similar results with dapagliflozin [51, 52], ipragliflozin [53], and luseogliflozin [54], and a recent Japanese study showed histopathological improvement with canagliflozin (25), suggesting that this beneficial effect could be a class effect. However, few studies used ^1^H-MRS, one of the most accurate and reproducible non-invasive techniques for NAFLD assessment in patients with obesity and T2D [55]. We observed a significant parallel decrease in visceral fat accumulation (− 6%) in the EMPA group compared to placebo, which is in agreement with a recent meta-analysis [48]. Taken together, these results suggest that SGLT-2 inhibitors could have a therapeutic effect on NAFLD and visceral adiposity in patients with T2DM.

### Twelve weeks of empagliflozin treatment did not change high-energy phosphate metabolism in humans

In this study, we also tested the myocardial energetics hypothesis in humans in vivo and investigated whether an increase in plasma ketones by empagliflozin was associated with an increase in rest cardiac PCr/ATP using ^31^P-MRS. Remarkably, we found no effect of empagliflozin on PCr/ATP ratio, despite a significant increase in blood KB levels, while PCr/ATP was significantly decreased in T2D patients compared to healthy controls (data not shown). To our knowledge, although we did not assess myocardial substrates disposal, this is the first study to report the effect of an SGLT2 inhibitor on PCr/ATP in humans. In isolated hearts of healthy mice, Uthman et al*.* [56] showed no effect of empagliflozin, dapagliflozin, or canagliflozin on PCr/ATP, but found inhibition of cardiac NA + /H + exchanger and significant reduction in cardiac cytosolic Na + concentration, suggesting another mechanism by which SGLT2 inhibitors could exert beneficial effects in heart failure. By contrast, Santos-Gallego et al*.* [49] reported increased myocardial ATP content and enhanced myocardial work efficiency in empagliflozin-treated nondiabetic pigs. Abdurrachim et al*.* [57] showed a 45% increase in cardiac PCr/ATP in EMPA treated *db/db* mice compared to placebo-treated *db/db* mice. Verma et al*.* using isolated working heart from *db/db* mice, demonstrated that empagliflozin increased cardiac ATP production by 30% and prevented heart failure. This effect was due to an increase in glucose and fatty acid oxidation, but with no change in ketone oxidation, suggesting that empagliflozin enhances the cardiac energy pool by increasing fuel supply to the failing heart, more than supplying the heart with a more efficient source of fuel [4, 58]. In our study, significant increase in hematocrit and EPO was observed in the EMPA group compared to placebo, possibly contributing to enhanced myocardial tissue oxygen delivery. Altogether, our results do not support an early change in myocardial energetics at rest in overweight T2D patients with normal LVEF treated with empagliflozin for 12 weeks. Whether empagliflozin can improve cardiac energetics in the context of increased workload such as was observed in obese subjects with weight loss needs to be evaluated [59].

The strengths of this study lie in the use of preclinical and clinical models and a randomized controlled trial design with concomitant measurement of several ectopic fat stores using novel multiple cardiac imaging modalities.

### Limits

We also acknowledge some limitations, such as that myocardial energetics was only evaluated in humans, in a subgroup of patients, only at rest, and in an exploratory analysis. An effect of empaglifozin on myocardial energetics during stress or exercise or later on can therefore not be excluded. We acknowledge that the intervention period may have been too short to observe a significant change in epicardial, myocardial fat and myocardial energetics. Longer interventional studies are now needed to draw definite conclusions on the effect of empagliflozin on cardiac ectopic fat stores. However, the sensitivity of ^31^P-MRS to detect changes in tissue energetics was high, as we were able to measure a significant decrease in PCr/ATP in T2D versus healthy controls. The sample size was perhaps also insufficiently powered to see an effect, but these results can serve to design future prospective studies. We could not assess PCr/ATP in mice for want of a ^31^P surface heart coil adapted to small animals. We also did not assess pancreatic fat in mice because of the well-known difficulty of delineating the pancreas in rodents with MRI. Finally, we did not assess myocardial substrates disposal in this study, but a recent study showed a > 50% decrease in myocardial glucose uptake after 4 weeks of treatment with empagliflozin in T2D individuals [18].

In conclusion, this randomized controlled trial showed that empagliflozin promptly reduced liver and visceral fat compared to placebo without changing cardiac ectopic fat or myocardial energetics. This study combined multiple novel cardiac imaging modalities such as ^1^H-MRS and ^31^P-MRS for ectopic fat and cardiac metabolism quantification, which are innovative, but validated and robust non-invasive MRI techniques.

## Conclusions

The study suggests that SGLT-2 inhibitors are useful agents for improving NAFLD, which often coexists with type 2 diabetes. To investigate whether SGLT-2 inhibitors also lead to improvement in steatohepatitis NASH and/or liver fibrosis would require randomized histopathological studies. In this study, no effect of empagliflozin on epicardial, myocardial, pancreatic fat or PCr/ATP ratio was evidenced at 12 weeks. The absence of any effect of empaglifozin on pancreatic and cardiac steatosis strengthens the tissue-specific mobilization of ectopic fat stores with therapeutic interventions. Further experimental and clinical studies are now needed to elucidate how SGLT2 inhibitors exert these impressive cardiovascular effects.

## Supplementary Information

**Additional file 1.** Supplementary material and methods for animals.

## Data Availability

The datasets used and/or analyzed during the current study are available from the corresponding author on reasonable request.
