# Supplementary material for: Effect of empagliflozin on ectopic fat stores and myocardial energetics in type 2 diabetes: the EMPACEF study
Source: Cardiovasc Diabetol. 2021 Mar 1;20:57. doi: 10.1186/s12933-021-01237-2 (PMC7919089; doi:10.1186/s12933-021-01237-2)
Supplement: Supplementary file 1 — Additional file 1. Supplementary material and methods for animals. [file 12933_2021_1237_MOESM1_ESM.docx]

**Additional file 1: appendix**

**Animals and experimental procedure**

All animal procedures were approved by the ethics committee of Aix-Marseille University and complied with the European Convention for the protection of animals used for experimental purposes.

Thirty-four C57BL/6 8-week-old male mice (Janvier, France) were housed in a controlled environment under standard laboratory conditions (12 h–12 h light-dark cycle, 22°C). Mice were randomly divided into: Control (*n* = 10) with standard diet (#U8224G10R, Safe Diets, France) and HFHS (*n* = 24) diet (#U8978P Version 0019, Safe Diets, France) as previously described (12) (Figure 1). At 4 weeks, metabolic status (intraperitoneal glucose tolerance test (IPGTT), glucagon, insulin and blood ketones) and MRI evaluation of ectopic fat stores (liver and myocardial, as rodents have no epicardial fat) were performed. The HFHS group was then randomly divided into two subgroups, EMPA (*n*= 12), with empagliflozin in water (30 mg/kg bodyweight + 0.1% DMSO (Sigma, USA)), and a placebo group HFHS (*n* = 12) with 0.1% DMSO in water. Metabolic status and MRI evaluation were performed after 4 and 12 weeks of treatment. At 24 weeks, mice were sacrificed, and tissues (heart, liver) collected.

**Mouse metabolic exploration**

Intraperitoneal glucose tolerance test (IPGTT) was performed after 6 hours fasting with a bolus of glucose (1 mg/g) at W0, W4 and W12. Blood glucose level was measured using a glucometer (AccuCheck Roche, Switzerland) before glucose injection (0 min) and at 15 min, 30 min, 60 min, and 120 minutes after injection.

**Biochemical analyses**

Blood ketones were assayed using a Freestyle Optium Neo (Abbott, USA) at W4 and urine glucose was assayed with urinalysis strips (Acon, USA) each time. At W12, insulin (Alpco, USA), glucagon (Mercodia, Sweden) and beta-hydroxybutyrate levels (Cayman Chemical, USA) were measured with serum samples according to the manufacturer’s recommendations. HOMA-IR in mice was calculated using the equation ((fasting glucose concentration × fasting insulin concentration)/405) as previously reported (47).

**qRT-PCR**

RNA was extracted from heart using the RNeasy RNA Mini Kit (Qiagen). Complementary DNA was synthesized using M-MLV reverse transcriptase kits (ThermoFisher). Reactions for qRT-PCR were prepared with sequence-specific primers and fluorescent Evagreen (Eurogentec). RPLP0 was used as housekeeping gene for normalized relative quantification of mRNA expression using the 2^–ΔΔCt^ method.

| Primer | Forward | Reverse |
| --- | --- | --- |
| *Bdh1* | GCTCCCAGGAAAAGCCCTAA | CATCTGCCTGACTGGCGTAG |
| *Bdh2* | CAACATGTCGTCTGTGGCCT | GGACTTGGTGAGACCGATCA |
| *Hmgcs2* | CTACCCGAGTGGTAACGCC | ACGCGTTCTCCATGTGAGTT |
| *Oxct1* | GCCAGCAACTTCATCAGTCC | AGATCCGCATCAGCTTCGTC |

**Oil Red O Staining**

After sacrifice, tissues (heart and liver) were embedded in OCT (Thermofisher) and cryoconserved. Cut sections of 12 μm were used for staining. Slides were left for 10 min at room temperature before staining with 0.5% Oil Red O solution (Sigma-Aldrich) for 5 min and washed with PBS. Counterstaining with 0.1% toluidine blue was performed for heart section. Stained slides were evaluated under a microscope with 10 random fields from each slide. Intensity was evaluated by ImageJ (NIH, USA).
